# Trehalose extends healthspan in *C. elegans*

**DOI:** 10.17912/W2RP4B

**Published:** 2016-10-28

**Authors:** Kristin J. Robinson, Kathyrn McCormick

**Affiliations:** 1 NemaMetrix, Inc,. 44 W 7th Ave., Eugene, OR 97401 USA.

**Figure 1.**
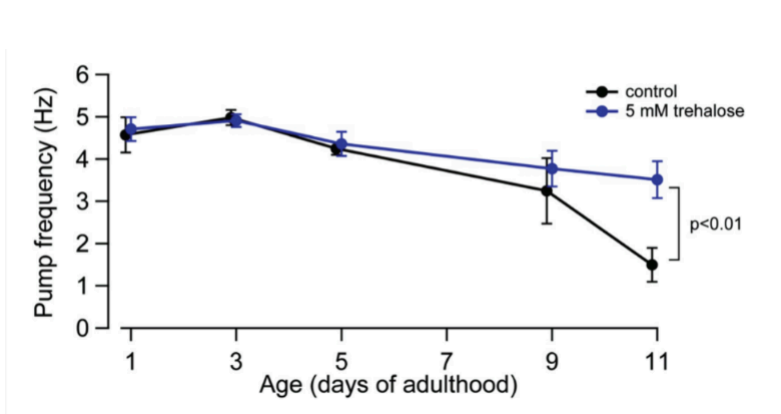


## Description

Adult worms were reared and tested in M9 medium plus 5 mM trehalose, which has previously been shown to extend lifespan and increase pump frequency late in life in *C. elegans* (Honda et al., 2010). Using an alternate electrophysiological readout of pumping, electrophayrngeograms (EPGs), we examined pump frequency at earlier timepoints than previously reported. Pumps were stimulated with 10mM 5HT in M9, recorded as EPGs in a microfluidic device, and analyzed using NemAnalysis software (NemaMetrix). Pump frequency in trehalose-treated animals was significantly higher in 11-day adults than in controls of the same age that were reared in parallel and tested on the same day (p<0.01; 1-tailed Mann-Whitney U-test; n = 5-8 worms at each age in each condition). This finding confirms, by an independent method, previously reported data (Honda et al., 2010).

## Reagents

Molecule: trehalose

Strain: N2
